# Supplied Blood in Extremis—A Killer of Septicæmia and Syphilitic Virus

**Published:** 1897-04

**Authors:** W. H. Parsons

**Affiliations:** Omaha, Neb.


### Abstracts and Selections.

### Supplied Blood in Extremis.—A Killer of Septicaemia and of Syphilitic Virus.

CASES BY DR. W. H. PARSONS, OMAHA, NEB.

I.

Miss B., age 16, of Lincoln, Neb., was admitted to hospital in Kansas City, Mo., June 19th, 1891. Laparotomy for ovarian cyst was performed on June 12th. She was anæmic in the extreme when admitted, and generally in bad condition for an operation, but the case demanded immediate relief, and the operation was deemed particularly successful; but the low vitality and extreme nervous irritability of the patient gave no promise of a favorable outcome.

Shortly after the operation the stomach became so irritable that all nourishment and even cold water were rejected. The temperature and other grave symptoms indicated sepsis. On June 18th, the date of my first visit to the hospital, the patient's life was despaired of, and the last rites of the church were being administered at the time of my arrival. Dr. G., the surgeon in charge, kindly gave me a history of the case. Rectal feeding had already been tried with unsatisfacthry results, beef tea and milk having been used. At my earnest request I was permitted to test the value of the blood treatment, the doctor saying at the time that the patient would not live forty-eight hours. Bovinine, one ounce, sterilized water, one ounce, pancreatine, five grains, raised to a temperature of 100 degrees F., were employed, and forced high up into the rectum. This was retained, and the same dose was repeated after an interval of two hours. After eight hours the distress and painful retching subsided, and if food was not alluded to the stomach remained tranquil. For twelve days the only nourishment administered was bovinine every three hours day and night, and by this process of nutrition alone, the vitality of the patient was restored, so that at the end of that period she sat up in bed, and for the first time since the operation expressed a wish for food. On July 3d, this moribund girl was pronounced convalescent.

II.

In St. Louis, a lady had pricked her thumb with some poisonous product, and blood poisoning in its most virulent form supervened, and in spite of the best efforts of several leading surgeons, the case came to a point where amputation at the shoulder seemed the only alternative. The hand and arm were swollen to their fullest capacity, and honey-combed with scores of sloughing ulcers. Upon my advice the hand and arm were dressed six times each day, after having been thoroughly cleansed, with pure bovinine, the ulcers being packed with soft lint saturated with the same, and the entire arm and hand dressed with it. In thirty hours a change was manifest, and in sixty hours healthy granulations began to appear, diseased tissue to slough out, and in twelve days her hand and arm were as good as new.

III.

A man in St. Joseph, Mo., wounded himself in the hand while dressing dead hogs at the yards. Blood poisoning set in in earnest. In six days all dressings, etc., had failed, and amputation was suggested. I was in the attending surgeon's office when he related the case to me. I suggested wrapping the arm and hand in bovine blood, changing every four hours. In twelve hours the change was so marked that the doctor sent for me to see the case. In four days he was well. The doctor thanked me, as did the man, who was about to lose his arm, and probably his life.

IV.

A man in St. Joseph's Hospital, Oneota, had his arm smashed in a railroad accident; the fractures were compound and badly comminuted, and in a few days an erysipelatous condition set up, which threatened his life. I was in the hospital, and the attending surgeon, an old friend of mine, Dr. E. W. Lee, chief surgeon of the B. & M. railroad, called me to view the case. It was truly desperate. I advised taking off all dressings, put the arm on a pillow, cleanse it thoroughly with hot bichloride, and wrap the entire arm in pure bovinine. After some hesitation it was done, and in four days the condition had so far changed as to allow the arm to be put back into the dressings. Another life saved, and another victory for blood.

V.

Soft chancroid involving the glans and prepuce. The soft ulcer had been doing its work for four weeks; appeared almost malignant; various dressings had failed, such as iodoform, etc., etc. This ulcer was packed in pure bovinine and soft lint, changed every two hours the first three days, then every four hours. In thirty six hours the diseased tissue sloughed out, healthy granulations set up, and in ten days he was well. This, in brief, is my experience along new lines (that is, new to me).

814 N. 23d st., Omaha, Neb., Nov. 22, 1895.

